# Review on Effectiveness of Primary Prophylaxis in aPLs with and without Risk Factors for Thrombosis: Efficacy and Safety

**DOI:** 10.1155/2014/348726

**Published:** 2014-04-17

**Authors:** Nahid A. Qushmaq, Samar A. Al-Emadi

**Affiliations:** ^1^King Abdullah Medical Complex-Jeddah (KAMCJ), Al-Ameer Nayef Street, North Obhur, P.O. Box 55659, Jeddah 21544, Saudi Arabia; ^2^Department of Medicine, Rheumatology Section, Hamad Medical Corporation, P.O. Box 3050, Off Al Rayyan Road, Opposite Lulu Center, Doha, Qatar

## Abstract

*Context*. Antiphospholipid antibodies syndrome is an autoimmune disorder that is characterized by the association between presence of antiphospholipid antibodies and risk of thrombosis and/or pregnancy morbidity. *Objectives*. To systematically review the evidence for primary prophylaxis in patients with antiphospholipids antibodies syndrome or APS with or without other traditional risk factors of thrombosis when they did not have any thrombotic event yet. *Methods*. PubMed, the Cochrane Library, and Allied Health Literature were searched for studies that examined the efficacy and safety of primary prophylaxis in aPL patients from 1990 to February 2013. We examined literature looking at patients with aPLs with other risk factors for thrombosis and aPLs with no additional risk factors for thrombosis. *Conclusion*. We concluded that, in patients with aPLs, primary prophylaxes with HCQ and aspirin have been observed to reduce the frequency of thrombotic events in the case of asymptomatic aPL-positive patients with SLE. We also in this study concluded that LDA was effective in patients with autoimmune diseases. Independent cardiovascular risk factors include autoimmune defects such as SLE, rheumatoid arthritis, and atherosclerosis, where overall venous thrombosis will be induced by systemic inflammation. This review concludes that HCQ is an effective primary approach when compared to aspirin.

## 1. Introduction

A commonly occurring systemic autoimmune disorder, antiphospholipid syndrome (APS or Hughes syndrome), is characterized by arterial and/or venous thrombosis, pregnancy morbidity, or recurrent miscarriage. It is also characterized by thrombocytopenia, increased levels of antiphospholipid antibodies (aPL), such as positive lupus anticoagulant (LAC), which is the most common, and/or anticardiolipin antibodies (aCL) [1] and *β*_2_-glycoprotein I antibodies [2]. The mechanism of actions proposed for thrombosis includes endothelial injury mediated by free radicals, coagulation malfunction, and compliment activation [3, 4]. Two hit hypotheses have also been proposed for the occurrence of thrombosis. For the first hit, a prothrombotic state is created by the presence of antiphospholipid antibodies. The second hit happens after a prolonged sustenance of antiphospholipid antibodies, following which the thrombosis condition becomes evident [5, 6]. However, not all individuals with high levels of aPL develop thrombosis [3], and hence it cannot be assumed that all antiphospholipid antibodies are thrombogenic [7, 8]. Various clinical studies have shown that aPL-related thrombosis is accompanied by irreversible functional loss of organs and lethality in systemic lupus erythematosus (SLE) [9].

APS was first detected in individuals suffering from SLE and was later recognized in patients with other autoimmune diseases, although at a lower frequency compared to SLE patients. Most of the case studies have shown that patients who have elevated levels of antiphospholipids and were affected by thrombosis also had other common habitual risk factors, such as smoking and a high BMI; allied risk factors included hyperlipidaemia, hypertension, high triglycerides, and type II diabetes [10–13]. While it is still unclear if the presence of antiphospholipid antibodies is an additional risk factor for individuals who are not affected by autoimmune disorders, it can be hypothesized that the extent of endothelial injury intensified over time caused by the abovementioned common risk factors is reflected in increased levels of antiphospholipid antibody and the presence of LAC [4, 14]. Nevertheless, this does not provide a clear understanding of the role of traditional risk factors and thrombosis. Moreover, it can also be argued that APS is not dependent on any other disease that may already be present.

### 1.1. Background

Patients who show antiphospholipid antibodies should be immediately treated to prevent thrombosis events. These patients will fall into two categories: those who have experienced a thrombosis event previously and those who have not. If the patient has already had a thrombosis event, it is called secondary thromboprophylaxis. The second category will include asymptomatic patients, SLE patients, and female patients who have obstetric antiphospholipid syndrome (primary thromboprophylaxis).

Considering that aPL-related manifestation of the syndrome is associated with a high morbidity and lethality rate, preventing the incidence of primary and secondary thrombosis is imperative [15, 16]. At present, patients affected with thrombosis as a result of high aPL levels are recommended for secondary prophylactic measures to reduce mortality rates. However, the time, methods, and target of drugs are highly debated [17–19]. Nevertheless, several effective therapeutic methods have been documented in clinical studies. It has been understood that multiple pathological processes are involved in APS while analyzing the nature of the clinical manifestations of this disease. Most of the existing treatments focus on the anticoagulation process. While 70% were found to show positive results, this leaves a substantial number of patients with no effective treatment. Furthermore, patients may experience bleeding complications with conventional anticoagulation therapy (2-3%) [20]. The optimal treatment in APS patients resistant or intolerant to long-term anticoagulation remains unclear. Recent improvement in the understanding of the pathogenic mechanisms, including aPL-induced activation of platelets, endothelial cells, monocytes, complement, and coagulation cascade, has led to the identification of potential targets and future therapies for APS.

Such evidence-based therapeutic methods are not well known for primary thrombosis; hence, controlled and potential clinical trials are underway [15, 21, 22]. Due to a lack of evidence-based therapeutic measures, patients with SLE and high aPL levels not affected by thrombosis are generally not recommended for any prophylactic measures, although some patients are administered aspirin and hydroxychloroquine (HCQ) [23, 24]. Aspirin is commonly prescribed for the prevention of primary thrombosis in populations exposed to traditional risk factors and secondary venous thrombosis [25]. Again, the preventive ability of aspirin in aPL patients has not been proven yet due to conflicting results obtained in clinical studies. Further, HCQ has been used in some clinical trials as a therapeutic drug for SLE patients and for APS in animal models [26–28]. In fact, there has been a lack of conclusive evidence as to whether to choose aspirin, HCQ, or a combination of these two in patients positive for aPLs with other risk factors for thrombosis and with no additional risk factors.

Although plenty of data is available on APS, evidence-based studies on the efficacy of therapeutic methods are not sufficient. There are no extensive studies on the effectiveness and safety of primary prophylaxis in aPL patients who are identified with other risk factors for thrombosis or aPL patients who have no risk factors for the same. However, the efficacy of primary prophylaxis in these two groups has been analyzed and compared in this study. Following this comparison, this paper represents and discusses the benefits in considering HCQ for primary prophylaxis. It also examines the literature on HCQ and compares the results obtained from the trials to arrive at a conclusion. Through this systematic review, we intend to collate information on the effectiveness of treatment methods available for the prevention of primary thrombosis in aPL patients with and without risk factors. With this background, the study developed the following research questions.

## 2. Research Methodology

The present section describes the research methodology adopted for the research entitled, “Effect of primary prophylaxis in preventing thrombosis in aPLs patients.”

### 2.1. Formulating Review Questions

The first step with either methodology is to develop a question, which is paraphrased as “formulating review question” for systematic reviews (Table 1).

This program of research is defined by two key research questions.

1. Is primary prophylaxis safe and effective in preventing thrombosis for patients both with and without other risk factors for thrombosis in antiphospholipids syndrome aPLs?
2. What is the best treatment to prevent recurrent thrombosis in aPL patients with and without additional risk factors for thrombosis and venous and arterial events not fulfilling and fulfilling criteria for APS?

The review will predominantly focus on primary prophylaxis as a treatment for patients with elevated aPL, with and without additional risk factors for thrombosis.

### 2.2. Eligibility Criteria

For the systematic review, this next step is known as “defining exclusion and inclusion criteria”.

#### 2.2.1. Search Strategy and Inclusion/Exclusion Criteria

PubMed, the Cochrane Library, MEDLINE, and Allied Health Literature were searched for studies that examined the efficacy and safety of primary prophylaxis in aPL patients. To reflect current aPL practices and techniques, the literature search was limited to studies published from January 1990 to February 2013. The main keywords used for searching in all of the databases were as follows.* Searches were conducted using a combination of three terms from the following sets: (1) “antiphospholipid syndrome,” “antiphospholipid antibodies,” “thrombosis,” and/or “beta 2-Glycoprotein I”; with (2) “thrombosis not children,” “not review,” “not recurrent,” “antiphospholipid syndrome,” or “lupus anticoagulant” or “primary prevention,” “primary prophylaxis,” or “primary therapy” or “drug therapy,” “antiphospholipid syndrome/therapy,” “antibodies”; and (3) “thrombosis therapy.”* In addition to this, the references that were mentioned in the articles chosen were reviewed in the hope that they might reveal further insights into this subject area.

Studies assessing the effect of primary prophylaxis in preventing thrombosis in antiphospholipid syndrome aPLs were explored. Subsequently, studies that assessed efficacy and safety levels of primary prophylaxis, followed by identification of the best treatment to prevent recurrent thrombosis in aPL patients and venous and arterial events not fulfilling and fulfilling criteria for aPL, were included. The review focused only on a main objective of primary prophylaxis as treatment for individuals with aPL who had not yet suffered any thrombosis. Finally, studies in a language other than English were excluded from our review. Our search strategy was used to identify a set of potentially relevant studies. Using predetermined selection criteria, each study identified during the search process was independently assessed by the researcher to determine its suitability for data extraction.

## 3. Results

In this study, a total of 101 citations were retrieved and reviewed for inclusion. A total of 25 studies were identified, and 2 further studies were identified by manual review of the reference list of relevant review articles. However, only 21 were found to be eligible. A total of 22 studies (randomized controlled trials = 7; retrospective = 7; prospective = 8) were therefore included in this review.

### 3.1. RCTs

Among the articles identified, only one RCT (*n* = 1) was eligible for the present study. The study compared the efficacy of aspirin 81 mg daily versus placebo for the prevention of thrombotic complications among 98 asymptomatic patients with persistently positive aPL. The aPL was measured on two occasions, 6 weeks apart. The study participants were predominantly female, of whom more than 60% had SLE. In a separate parallel observation study, patients who were aPL-positive and taking aspirin were declined randomization and followed prospectively. The acute thrombosis incidence rates in aspirin-treated subjects were 2.75 per 100 patient-years and, for placebo-treated, 0 per 100 patient-years (hazard ratio: 1.04; 95% CI: 0.69–1.56, *P* = 0.83); in the observational study, the incidence rates were 2.70 per 100 for aspirin treated and 0 per 100 for not treated. However, this study was terminated due to an unexpectedly low rate of thrombotic events (arterial or venous) (*n* = 3) occurring in the aspirin group and none in placebo. Although this study observed infrequent outcome events and was limited by the small number of patients enrolled, in the patients with persistently positive aPL aspirin was not effective in comparison to placebo for the primary prevention of thrombotic events. Thus, the study revealed no benefit from low-dose aspirin for primary thrombosis prophylaxis [29].

Arterial and venous thrombotic manifestations can be prevented by using a prophylactic treatment with aspirin in the case of SLE patients. Using a Markov decision analysis, Wahl et al. [30] show this applies particularly to patients with positive aPL. The study also discovered that patients who tested positive for aCL or LA, or patients who had lupus, were found to have prevented bleeding episodes by keeping a good balance between thrombotic events. However, another clinical trial (APLASA) that was conducted on patients with SLE showed that there were not many differences among aPL-positive patients treated with either low-dose aspirin or placebo [29]. The authors noted that the zero event rate in the case of the placebo group can be attributed to a number of factors, such as the inclusion of individuals (over 40%) with a low-risk aPL profile, a short followup, and good control of vascular risk factors.

In the case of asymptomatic individuals with aPL, the probability of thrombosis is lowered when aspirin or HCQ is used [10]. The additive clinical thrombotic risk factors, along with the likely preventive treatments, were analysed in a cross-sectional study that surveyed 77 APS patients. The results from the study were compared to the results that were obtained by patients who were asymptomatic aPL-positive and who had no history of pregnancy morbidity or vascular thrombosis. The analysis of the results indicated that pregnancy (*P* = 0.005) and surgical procedures (*P* = 0.04) were significantly more frequent in group A, whereas aspirin (*P* < 0.001), HCQ (*P* < 0.001), and corticosteroids (*P* = 0.002) were used extensively more frequently in group B. Surgical procedures and pregnancy, along with thrombocytopenia, raised the risk of an event in the case of women. The study also showed an increased risk of hypertension along with a number of other factors, especially in the case of arterial thrombosis; however, these factors were not shown to be associated with venous thrombosis. The risk of thrombosis was also increased by certain traditional factors that were similar among various groups. Hypertension and smoking were associated with arterial events. However, there is still a lack of sufficient research that has dealt with the question of whether or not the efficacy of primary thromboprophylaxis in this group of patients with SLE and aPL increases for the combination therapy of antimalarials plus low-dose aspirin.

Another research trial by Finazzi et al. [31] evaluated 109 patients who suffered from antiphospholipid antibody syndrome. The trial attempted to determine the optimal intensity of oral anticoagulation for the prevention of recurrent thrombosis. In order to determine conclusively that intensive anticoagulation is better than standard treatment for preventing symptomatic thromboembolism without increasing the bleeding risk, the study gave two possibilities. One was to use either standard antithrombotic therapy (warfarin, INR range 2.0-3.0 in 52 patients or aspirin alone, 100 mg day(−1) in 3 patients); the other was to use a high-intensity warfarin (INR range 3.0–4.5, 54 patients) with a follow-up median time of 3.6 years. The study's results showed that when conventional treatment was being given to the patients, recurrent thrombosis could be seen in 3 out of 55 patients (5.5%). In the case of patients who were given high-intensity warfarin, recurrent thrombosis could be seen in 6 of 54 patients (11.1%) [hazard ratio for the high-intensity group, 1.97; 95% confidence interval (CI) 0.49–7.89]. In addition to this, out of the 15 patients who received high-intensity warfarin, major bleeding was observed in 2 of them while the rest showed minor bleeding. In the case of patients who received conventional treatment, 8 patients showed major bleeding (hazard ratio 2.18; 95% CI 0.92–5.15). Thus, it is clearly evident from the trial results that high-intensity warfarin is not a better treatment than conventional treatment when it comes to preventing recurrent thrombosis in patients with APS, as high-intensity warfarin-based treated patients were more prone to minor hemorrhagic complications.

In a randomized control study, one group of asymptomatic antiphospholipid antibody carriers was administered a low-dose aspirin and the other group was given a placebo [27]. The results of this study have shown no considerable differences between these two groups, but the placebo group showed 0% of incidence for thrombosis; hence there was a necessity to under-power this trial for analyzing the role of aspirin. As a result of the low possibilities for risks, followup has become simple, vascular risk factors can be controlled effectively, and the immunological status of the patients can be preserved. However, the study did not provide the exact count of the lupus anticoagulant and obstetric antiphospholipid patients. On the contrary, the effect of aspirin on asymptomatic antiphospholipid antibody carriers who have SLE has been shown in the outcomes of the observational studies [10, 32, 33]. As a result of the benefits associated with low-dose aspirin, the patients who have SLE or anticardiolipin or both have been treated with the combination of low-dose aspirin and HCQ by the experts. Low-dose aspirin has been found to be useful in providing long-term treatment for the female patients who have obstetric antiphospholipid syndrome. However, this treatment will be recommended based on the immunological profile of the patients. It is necessary to individualize the treatment for healthy carriers of antiphospholipid antibodies as well as for patients who have a wide range of antiphospholipid antibodies such as lupus anticoagulant. In addition, the patients who have antiphospholipid antibodies should be treated by giving considerable importance to other vascular risk factors. It has been understood from the literature that both anticoagulation and extremely concentrated steroids can be used for the treatment of patients with less severe cases of CAPS. Contrastingly, if the cases are extremely severe, it is necessary to use plasma intravenous immunoglobulins or plasma exchange [34]. These treatments were found to be life-saving in 65% of severe cases.

Rand et al. [35] examined the influence of HCQ on the synthesis of aPL-*β*_2_GPI complexes on phospholipid bilayers using ellipsometry and atomic force microscopy (AFM). The interaction between aPL-*β*_2_GPI complexes and THP-1 (human acute monocytic leukemia cell line) monocytes can be prevented with 1 *μ*g/mL concentration of HCQ. The interaction between the individual proteins and bilayers can also be prevented by the same means. If the HCQ protein is dialysed against a buffer, then the aforementioned reductions in binding can be reversed. It is also possible to obtain statistically significant reductions of clinical antiphospholipid assays through HCQ. Hence, the synthesis of aPL-*β*_2_GPI complexes has been found to be reduced with HCQ. The results of this study support the anticoagulant nature of HCQ.

Willis et al. (2012) studied the interactions of HCQ with proinflammatory/prothrombotic markers in 35 SLE patients who were examined in a multiethnic, multicenter cohort (LUMINA) using SLAM-R scores. A multiplex immunoassay assessed the level of interferon- (IFN-) *α*2, interleukin- (IL-) 1*β*, IL-6, IL-8, inducible protein- (IP-) 10, monocyte chemotactic protein-1, tumor necrosis factor- (TNF-) *α*, and soluble CD40 ligand (sCD40L) levels. A positive correlation between SLAM-R scores at baseline and levels of IFN-*α* (*P* = 0.0546) was observed. Hydroxychloroquine therapy resulted in a significant decrease in SLAM-R scores (*P* = 0.0157), and the decrease in SLAM-R after HCQ therapy strongly correlated with decreases in IFN-*α* (*P* = 0.0087).

### 3.2. Retrospective Studies

Of all the articles that were researched, 6 studies were found to be relevant to the current research study. Numerous risk factors such as SLE-related factors and traditional or aPL-profile factors were evaluated by a study conducted by Tektonidou et al. (2009) [36] along with primary thrombosis prevention among 144 SLE patients. The selected patients were divided amongst those that did or did not have aPL, and the follow-up times were 104 and 112 months, respectively. In the case of aPL-positive patients, the study demonstrated that 20.1% of them showed high thrombosis rates and about 7.6% (*P* = 0.003) of the negative aPL patients showed high thrombosis rates. In the case of aPL-positive patients, a low dose of aspirin helped in protecting the patients against thrombosis (HR per month 0.98, *P* = 0.05). Similarly, the duration of hydroxychloroquine in both aPL-positive (HR per month 0.99, *P* = 0.05) and aPL-negative patients (HR per month 0.98, *P* = 0.04) also appeared to have protected the patients against thrombosis. Patient records were obtained retrospectively from their previous medical records, even if the study was longitudinal in nature. The final results may not be generalizable as the entire sample population was Caucasian. Even so, the results from the study are quite beneficial as the data relates to a number of SLE patients with high-risk aPL. It is also relevant to the evaluation of coexisting non-aPL thrombosis risk factors and the age- and gender-matched control group used, and it offers the opportunity to have detailed information on the patients' characteristics during a regular followup.

Another study by Ruffatti et al. [13] evaluated the overall risk factors associated with a first thrombotic event in aPL-positive carriers in people 18–65 years of age; it also explored the effectiveness of prophylactic treatments. The study evaluated 370 people that were analysed retrospectively for a mean (SD) followup of 59.3 (45.5) months. During the follow-up checks, 30 patients (8.1) developed a first thrombotic event. Some of the risk factors identified were hypertension and medium/high levels of IgG anticardiolipins. Thromboprophylaxis during high-risk and long-term periods was significantly protective. In the case of asymptomatic aPL carriers, once again medium/high titres of IgG aCL along with hypertension were determined to be risk factors, and it was also determined that primary prophylaxis is protective.

The study by Mok et al. [37] had a very diverse sample group, with a total of 625 patients, among whom 258 were Chinese, 140 were African American, and 227 were Caucasian. The study primarily sought to compare the incidence and risk factors associated with thromboembolic events in systemic lupus erythematosus (SLE). In the case of 83 patients, the study made observations over a period of 3,094 patient-years, 40 venous events, and 48 arterial events. The incidence of arterial thromboembolism was 16/1,000 patient-years, and the incidence of venous thromboembolism was 13/1,000 patient-years. Sixty months from the time of diagnosis of SLE, the study discovered that 8.5% of the Chinese were at risk of arterial events, followed by 8.1% of African Americans, and 5.1% of Caucasians. Similarly, the study also determined that 60 months from the time of diagnosis of SLE, the risk of venous events was 3.7%, 6.6%, and 10.3%, respectively (*P* = 0.008 for Chinese versus Caucasians, by log-rank test). Arterial events were predicted in a Cox regression model with the help of Chinese ethnicity, serositis, low levels of high-density lipoprotein (HDL) cholesterol, and oral ulcers. Similarly, venous events were predicted in a Cox regression model with the help of non-Chinese ethnicity, hemolytic anemia, low levels of HDL cholesterol, obesity, male sex, renal disease, and antiphospholipid antibodies. Thus, some of the differences and factors associated with arterial and venous thromboembolism in patients with SLE cannot be understood or explained. This indicates that more research is required in this field.

aPL-positive patients who have no symptoms of SLE have been assessed for the effect of HCQ on AnxA5 resistance assay (1997). Comparing the results of AnxA5 resistance assay before and after the administration of HCQ is the major aim of this study. The results from other tests such as LA test, aCL ELISA, ELISA, and anti-domain-I *β*_2_GP1 ELISA) have also been compared. This study recommends the administration of HCQ for treating recurrent APS.

Broder and Putterman [38] identified a link between HCQ aPL and LAC levels in 90 SLE patients who are older than 21. Here, the major outcome variable is LAC+ and/or at least 1 aPL ≥ 40 U [immunoglobulin A (IgA), IgG, or IgM]. Nineteen percent of the study population showed persistent LAC+ and/or at least 1 aPL ≥ 40 U. However, this level was found to be low with the administration of HCQ (OR 0.21, 95% CI 0.05, 0.79, *P* = 0.02).

### 3.3. Prospective Studies

The current study analysed clinical and analytical findings in 404 individuals among aPL-positive patients with SLE and included a followup for 36 months. The sample population was grouped into two categories:

1. asymptomatic carrier of aPL (*n* = 178);
2. patients with primary or secondary antiphospholipid syndrome.

Dicumarine was used to treat patients with APS and an international normalized ratio at or near 3.0 was targeted. Meanwhile, when the patients were at higher risk of thrombosis without treating asymptomatic carriers, then specific prophylaxis with low-molecular weight heparin or aspirin was used. The study revealed that 31% of the patients with APS showed arterial thrombosis and/or 46.9% of the patients with APS showed venous thrombosis or feta loss (51.8%). According to (2004) [39], aspirin or low-molecular weight heparins were found to be the best treatments for APS patients who suffered from thrombotic complications.

A study by Tarr et al. [32] outlined and grouped all of the risk factors associated with thrombotic events in 272 lupus patients. The study also carried out a 5-year followup with the patients. Three groups were classified at the baseline into an aPL+ group with 81 aPL-positive patients without clinical thrombosis; an aPL- group with 107 aPL negative patients; and a secondary antiphospholipid syndrome (APS) group with 84 aPL+ patients who met the Sapporo criteria. Another study by Hereng et al. [40] evaluated whether or not aspirin could provide primary prevention of antiphospholipid syndrome (APS) symptoms. In the study, a total of 103 individuals were taken as the sample, 75 of which were taking aspirin. The results indicated that, among aPL-positive patients, 16 had autoimmune thrombocytopenia (AIT), 20 had prolonged activated partial thromboplastin times, 11 had diverse diseases, 37 had systemic lupus erythematosus (SLE), and 19 had other connective tissue diseases. Furthermore, thrombotic events were experienced by 19 patients during the followup. Aspects such as HCQ use, clinical features, and biological parameters were similar across groups; however, there was a distinct difference regarding thrombosis (log-rank test; *P* = 0.02). Thrombosis developed among 4 of the 10 SLE patients who were not taking aspirin and among 3 of 27 patients who were taking aspirin (log-rank test; *P* = 0.03). Therefore, it can be stated that aspirin should be taken by aPL-positive SLE and AIT patients in order to prevent APS manifestations. More studies and trials are required to understand aspirin's prophylactic role.

Another study evaluated the risk factors for thrombosis in a large, multiethnic SLE cohort [41]. The study involved 1930 SLE patients from a variety of ethnic backgrounds, including Caucasians, African Americans, Asian Americans, and Hispanics. Some of the factors that were considered to be at risk for developing thrombosis were aPL positivity (OR 3.22, *P* < 10(−9), OR 1.26 per 5 years, and *P* = 0.027 × 10(−7)), immunomodulating medication use (OR 1.40, *P* = 0.011), nephritis (OR 1.35, *P* = 0.036), and smoking (OR 1.26, *P* = 0.011). SLE onset at younger ages was protective (OR 0.52 for age ≤20, *P* = 0.001), the use of HCQ was found to be the best treatment against thrombosis. Thus, the study confirms immunomodulating medication use, longer disease duration, history of nephritis, and older age at onset, along with aPL positivity, as risk factors for thrombosis in SLE.

Miscellaneous thrombosis risk factors, including aPL-profile, SLE-related, and traditional risk factors, have been analysed by Tektonidou et al. [36]. This study questions how thrombosis can be prevented in 144 patients with SLE (follow-up period 104 and 112 months) and those who do not have aPL. In aPL patients, thrombosis was predicted with male sex (hazard ratio [HR] 6.25, *P* < 0.01), LAC (HR 3.48, *P* < 0.04), and consistently positive aCL (HR 5.87, *P*  0.01). In aPL-negative patients, male sex (HR 7.14, *P*  0.03) and hypertension were markers. aPL-positive patients were found to be protected with the administration of LDA (HR per month 0.98, *P* < 0.05). In addition, both aPL-positive and -negative patients had been treated with HCQ (HR per month 0.99, *P* < 0.05) (HR per month 0.98, *P*  0.04), respectively.

In a cohort study conducted by Rubenstein et al. [42] on 1795 SLE patients who had aPL antibodies, the capability of HCQ in reducing thrombosis was proven.

The effect of HCQ on the AnxA5-RA in aPL-positive patients has been studied in a prospective study conducted by Rand et al. [43]. Here, AnxA5-RA is the primary measure whereas LA, aCL, a*β*_2_GPI, D-dimer, and anti-domain-I *β*_2_GPI antibody [aDI-*β*_2_GPI] are the secondary measures. This study involves the comparison of baseline data from 15 aPL-positive patients (mean age 43.3 ± 12.0 years [range 27–61], 10 [67%] female, 13 [87%] Caucasian; 10 had antiphospholipid syndrome, 5 asymptomatic aPL) and 7 aPL-negative SLE patients (mean age 40.0 ± 16.3 years [range 22–64], 7 [100%] female, 4 [57%] Caucasian). Positive AnxA5-RA, triple aPL-positive, double aPL-positive, and single aPL-positive were observed in 87%, 69%, 15%, and 15% of the aPL-positive patients, respectively. In this study, aPL-positive patients were identified with positive AnxA5-RA and aDI-*β*_2_GPI.

Vasculitis, premature atherosclerosis, and hypercoagulability are the major causes of thrombosis in SLE patients. In a Hopkins cohort study [19] of 100 patients, it was identified that thrombosis can be predicted with high anti-dsDNA and low C3, atherosclerosis (hypertension, hyperlipidemia, and elevated homocysteine), and antiphospholipid antibodies (lupus anticoagulant or anticardiolipin) and could be treated with HCQ.

Ruiz-Irastorza et al. [44] conducted a prospective study on a cohort of 232 patients to identify the effect of antimalarial drugs in preventing thrombosis in SLE patients through a Cox regression-multiple-failure time survival analysis model. In this study, the antimalarial drug was protective against thrombosis (HR 0.28, 95% CI 0.08–0.90), while aPL positivity (HR 3.16, 95% CI 1.45–6.88) and previous thrombosis (HR 3.85, 95% CI 1.50–9.91) increased the risk of thrombotic events. These results indicate the positive effect of antimalarials in preventing thrombosis in SLE patients.

## 4. Discussion

This systematic review comprehensively evaluated the efficacy, safety, and treatment methods for primary prophylaxis of therapeutic measures among patients positive for aPLs with other risk factors for thrombosis and aPLs with no additional risk factors for thrombosis. The study specifically compares the two groups of aPL with other risk factors for thrombosis and with no additional risk factors. Further, the study also focuses on primary prophylaxis to prevent thrombosis in antiphospholipid syndrome (APS). Moreover, by searching the literature and extant studies covering populations where hydroxychloroquine (HCQ) was effective and comparing them to other studies where HCQ was not effective as a primary prophylaxis, this review concludes that HCQ is an effective primary approach when compared to aspirin.

Along with HCQ, aspirin's effects as a preventive therapy have been analyzed in this review. Based on an assessment of the literature collected, it is clear that primary thrombosis prophylaxis strategies in asymptomatic aPL-positive with other risk factors for thrombosis and aPLs with no additional risk factors for thrombosis are limited. in a study conducted on antiphospholipids antibodies patients, an aspirin of 81 mg, was administered to asymptomatic, persistently aPL-positive individuals and another group was left on placebo (mean ± SD followup period 2.30 ± 0.95 years) [29]. Both of the groups were followed up prospectively, and the results confirmed that aspirin administration does not prevent thrombosis but that the net annual incidence was low.

Wahl et al. [30] showed that in Markov decision analysis when patients with SLE and aPL-positive are given a prophylactic treatment with aspirin, arterial and venous thrombosis is prevented. To add to this, a study performed by (2009) [3] also clearly demonstrated that incidence of thrombosis decreased in individuals with elevated aPL but was otherwise asymptomatic. Erkan et al. [16] also conducted a prospective study on the effect of low-dose aspirin on aPL-positive individuals without thrombosis. In subsequent study, the results suggested that, while low-dose aspirin did not prove beneficial, it did lower the overall annual rate of thrombosis incidence [29]. This study also included patients affected by other autoimmune disorders and asymptomatic individuals who did not contract autoimmune disease. Similar to SLE patients, these individuals also showed low incidence of autoimmune disease on administering low-dose aspirin [39]. Future followups showed that more than 50% of the patients who later developed thrombosis were diagnosed with SLE. Further, the study also revealed that 42% of the examined patients exhibited low-risk rates of thrombosis due to high aPL levels, which indicated lesser benefits from aspirin administration [45, 46]. The limitation of this study was that the follow-up time was only 2.3 years, which is brief compared to other related studies [47–49].

In a randomized clinical study, it was found that the overall incidence rate of thrombosis was 1.33 per 100 patient-years; for aspirin treated cases, the incidence rate was 2.75 per 100 patient-years, and, in the placebo group, the rate was 0 per 100 patient-years. Accordingly, in the observational study carried out, the overall incidence rate of thrombosis was 2.2 per 100 patient-years; in aspirin treated cases, the incidence rate was 2.70 per 100 patient-years and 0 per 100 patient-years in the placebo group of subjects. In this study, all patients (except one) had thrombosis risk factors along with the incidence of thrombosis. All of the above results indicate that persistently aPL-positive individuals receive little to no benefit from aspirin treatment for primary prophylaxis, but the overall annual incidence rate of acute thrombosis is minimized.

However, in the presence of other traditional risk factors, vascular disorders may occur. The limitations of this research are inadequate sample size, low frequency of the disease (which lowered the number of subjects studied), and the challenge in identifying asymptomatic patients. Despite these drawbacks, the APLASA study represents the first randomized study carried out with persistently aPL-positive subjects that was successful and consistent with many of the clinical and epidemiological studies conducted [22].

Not all asymptomatic patients with an aPL-positive diagnosis were given aspirin for three reasons.

- The unselected cases displayed a predicted thrombosis incidence rate of 1% patient-years.
- It is believed that aspirin could cause acute bleeding. Considering this level of thrombotic risk, the benefits did not outweigh the potential risks.
- The expected benefits of aspirin have been proven in at least one randomized clinical trial, even though there may have been few methodological limitations.

Various risk factors of thrombosis other than high aPL levels such as habitual risks (e.g., smoking) are alterable factors. Modifying these factors would be a manageable and safer approach to lower the rate of thrombosis. Pengo et al. [50] questioned: “Does aspirin prove to be beneficial for patients at particularly high risk of thrombosis including high risk for other autoimmune diseases and syndromes occurring with undocumented patterns of antibodies (triple positivity)?” This doubt remains to be confirmed with the help of more clinical trials. Currently, no substantial information is available to recommend aspirin as a primary prophylaxis treatment for thrombosis as the drug has demonstrated risk of bleeding [51]. However, the expert committee of European League Against Rheumatism (EULAR) has recommended guidelines for effective management of SLE. According to EULAR, low-dose aspirin can be recommended for primary thrombosis patients, despite the lack of substantial evidence [52]. Tektonidou et al. [36] have shown that HCQ effectively lowers the risk of thrombosis in both aPL-positive and -negative subjects. In addition, HCQ was administered in the 1970s and 1980s to treat deep vein thrombosis and pulmonary embolisms, which commonly affected patients who underwent hip replacement surgery. Several studies have shown results favourable to thrombosis prevention with the use of HCQ, even though its mechanism of action has been demonstrated only in animal models [26–28, 44]. HCQ has also been proven to have optimal effects on treating high lipid profiles [27, 53]. Although the protective effects of antimalarials in preventing thrombosis and its advantag against lupus activity are well documented, its beneficial effects in SLE patients are only minimally known [44, 54].

## 5. Conclusion

The past literature review indicates that the presence of aPL along with SLE increases the risk associated with thrombosis significantly [29, 36, 37, 55, 56]. Therefore, in the case of patients who are aPL-positive the highest percentage of thrombosis occurs in the subgroup with SLE [32, 36, 37, 39, 49, 57–62]. According to a study by Ruiz-Irastorza et al. [63], APS has been seen to have an adverse effect on the survival rate of SLE patients and to cause irreversible organ damage. In addition to this, no significant differences were discovered between low-dose aspirin and placebo in the APLASA clinical trials [29]. However, most of the clinical trials included patients who tested positive for SLE, and SLE itself is known to have its own set of influences over thrombosis; hence, the results obtained cannot be considered applicable to non-SLE populations [64–66].

Thromboembolic events can be effectively treated with or without antiphospholipid antibodies by means of HCQ in patients who have lupus [67–70] and in patients with aPL. Primary prophylaxes with HCQ and aspirin have been observed to reduce the frequency of thrombotic events in the case of asymptomatic aPL-positive patients with SLE. In a Markov decision analysis, oral anticoagulant therapy and observation, along with prophylactic aspirin, were found to be effective in reducing bleeding and incidences of thrombosis [30]. There is still a lack of sufficient research that has dealt with the question of whether or not the efficacy of primary thromboprophylaxis in this group of patients with SLE and aPL increases when both therapies (antimalarials plus low-dose aspirin) are combined. In the case of asymptomatic aPL carriers, medium/high titres of IgG aCL, along with hypertension, were determined to be the risk factors, and it was also determined that primary prophylaxis is protective. For the clinician who is faced with an aPL-positive patient with no history of pregnancy morbidity or thrombosis, it is better that an individual assessment of the patient's thrombotic risk be done. Aspirin by itself is still not considered to be the best treatment for primary thromboprophylaxis, considering that aspirin is a blood thinner [51]. Primary prophylaxis with aspirin, either alone or in combination with HCQ, is not advisable if the patient does not show any signs of SLE, cardiovascular, or thrombotic risk factors.

Further, the aPL carriers with autoimmune conditions will be treated in a different way. The annual incidence rate of thrombosis events in anti-CL-positive patients who have SLE was found to be 3.8% [49]. There has also been a study on the prophylactic role of LDA in aPL-positive SLE patients [30]. The possibilities for reducing the thrombosis events have been discussed in this study. In this study, it was proven that treatment-caused bleeding can be effectively treated with LDA. On the other hand, LDA was found to be effective in patients with autoimmune diseases. Independent cardiovascular risk factors include autoimmune defects such as SLE, rheumatoid arthritis, and atherosclerosis, where overall venous thrombosis will be induced by systemic inflammation. More than 1/3 of SLE patients' deaths were due to thrombosis events, and in these cases thrombosis can be predicted based on the status of aPL. In addition, female aPL-positive patients who have pregnancy morbidity will show high risks for thrombosis, yet they cannot fulfill the eligibility criteria for undergoing APS diagnostic procedures. The possibilities for vascular thrombosis after pregnancy can be reduced by using LDA. For example, fetal loss was observed in a single female aPL-positive patient only in a retrospective study. In this study, the control group showed 59% of incidence of thrombosis event, whereas the LDA group showed 10% of incidence [57]. It has also been identified from surveys that fetal loss will occur among the female patients who show strong aPL.

The body of research demonstrating effective therapies to manage aPL carriers is growing. For example, it has been identified that thrombosis events, common in patients with SLE, can be managed effectively with HCQ [32, 36, 41]. With its ability to inhibit the inflammatory cytokines such as TNF*α*, IL1, IL2, and IL6, HCQ can effectively prevent thrombosis. It is involved in the inhibition of TCR- and BCR-induced calcium signaling [71]. In addition, platelet aggregation and arachidonic acid release from stimulated platelets can also be inhibited with HCQ [72, 73]. Further, the interaction between anti-*β*_2_GP1 antibodies and the phospholipid bilayers [30], thrombus size, and total time of thrombus formation [32] can prevent thromboembolic events in SLE patients [40] and primary thrombotic events in asymptomatic aPL-positive patients [29] through HCQ. However, discovering the most effective therapeutic targets requires elucidation of the pathogenesis process involved in APS. To date, there is no in-depth knowledge on the advantages associated with therapeutic agents such as rituximab or alternative antiplatelet drugs; more researches and evidence are required.

## Figures and Tables

**Table 1:** Systematic review of primary prophylaxis in aPLs.

Category	Author, year	Study design	*N*	Number of aPL measurement	Comparison/intervention	Results/conclusion
RCT	Erkan et al., 2007 [29]	RCT with parallel prospective cohort	RCT = 98; cohort *t* = 74	≥2.6 weeks apart; observational 2.4 years	RCT: ASA 81 mg daily (*n* = 48) versus placebo (*n* = 50)	(i) HR: 1.04 (95% CI: 0.69–1.56) (ii) >60% of patients in RCT had SLE (iii) In comparison to placebo, ASA was not effective in preventing thrombosis
	Finazzi et al., 2005 [31]	Randomized trial	109	3.6 years	Conventional intensity or ASA alone *n* = 52 High-intensity warfarin *n* = 54	Conventional group HR 2.18 (95% CI 0.92–5.15) High-intensity group HR 1.97 (95% CI 0.92–5.15)
	Erkan et al., 2002 [10]	Cross-sectional	56	Not specified	Logistic regression analysis (ASA and/or HCQ use)	Probability of thrombotic event decreased in patients taking ASA +/− HCQ (HCQ only in patients with Connective tissue disease)—78% of patient had Connective tissue disease
	Wahl et al., 2000 [30]	Decision analysis			Observation ASA anticoagulation	In aPL-positive SLE patients, benefit of primary prophylaxis with ASA outweighs risk
	Willis et al., 2012 [74]	Multiethnic, multicenter cohort (LUMINA)	35	Not specified	Comparison of SLAM-R scores	Decrease in SLAM-R after HCQ therapy strongly correlated with decreases in IFN-α (P = 0.0087)
	Petri, 1996 [27]	Hopkins cohort study	100	12 months	Predictors for thrombosis in SLE patients and effects of HCQ on thrombosis	High anti-dsDNA and low C3, atherosclerosis (hypertension, hyperlipidemia, and elevated homocysteine)

Prospective	Girón-González et al., 2004 [39]	Prospective	178	>2.8–12 weeks apart	ASA 325 mg/d or LMWH daily during high risk situations*	All patients received thromboprophylaxis during high risk situations; no thrombotic events occurred
	Levine et al., 2002 [4]	Prospective study	22	Not specified	Effect of HCQ on the AnxA5-RA in aPL-positive patients	Positive AnxA5-RA, triple aPL-positive, double aPL-positive, and single aPL-positive were observed in 87%, 69%, 15%, and 15% of the aPL -positive patients
	Pierangeli et al., 1997 [26]	Prospective study	100	Not specified	Comparing the results of AnxA5 resistance assay before and after administration of HCQ	HCQ was found to be effective in reducing thrombosis events
	Cervera et al., 2009 [55]	Multicentre prospective study	1000	5 years	Morbidity and mortality in patients with APS	LDA can prevent thrombosis
	Tarr et al., 2007 [32]	Prospective	27281 aPL-positive	≥2.6 weeks apart	Prophylaxis (*n* = 52) versus observation (*n* = 29)**	Lower incidence of thrombosis in prophylaxis group versus observation group (1/52 versus 2/29 had stroke or TIA)
	Ruiz-Irastorza et al., 2006 [44]	Prospective cohort	232	1	Effect of antimalarial drugs in preventing thrombosis in SLE patients through a Cox regression-multiple-failure time survival analysis model	aPL positivity (HR 3.16, 95% CI 1.45–6.88)
	Rubenstein et al., 2006 [42]	Cohort study	1795	Not specified	Capability of HCQ in reducing thrombosis	HCQ was found to be effective

Retrospective	Hereng et al., 2008 [40]	Retrospective	103 (36% SLE or other Connective tissue disease)	64 months +/− 24 Unknown time interval between measurement	ASA (*n* = 75) versus observation (*n* = 28); HCQ in connective tissue disease	(i) In ASA group, lower frequency of thrombotic events observed (12% versus 35.7% overall; 11% versus 4% in SLE, particularly in the SLE or AIT subgroups of patients) (ii) 36% of patients had SLE
	Ruffatti et al., 2009 [13]	Retrospective	370 (35% SLE)	56.3 months	134 long-term prophylaxes with ASA 5 long-term prophylaxes with warfarin 48 prophylaxes only during period of high risk with ASA/heparin or both	Combination of high and long-term risk period of prophylaxis protective against thrombosis
	Tektonidou et al., 2009 [36]	Retrospective	288.144 aPL-positive	≥2–12 weeks apart	Adjusted survival analysis (ASA 80–100 mg/d, HCQ)	(i) HR per month: ASA 0.98 (95% CI: 0.96–0.99) and HCQ 0.99 (95% CI: 0.98–1.00) (ii) Duration of use of ASA and HCQ associated with decreased thrombosis
	Kaiser et al., 2009 [41]	Retrospective	1930.516 aPL-positive	1	Logistic regression analysis (HCQ use)	(i) OR 0.63 (95% CI: 0.48–0.83) (ii) HCQ protective against thrombosis
	Mok et al., 2005 [37]	Retrospective	83	11 years		HCQ intake showed lower risk for thrombotic events (OR 0.21 95% CI: 0.06–0.81)
	Broder and Putterman, 2013 [38]	Retrospective study	90	Not specified	Link between HCQ aPL and LAC levels	19% of the study population showed persistent LAC+ and/or at least 1 aPL ≥ 40 U
	Wallace et al., 1993 [28]	Retrospective	72	6 years	Logistic regression analysis (HCQ use)	*P* < 0.05
